# The evaluation of the role of medial collateral ligament maintaining knee stability by a finite element analysis

**DOI:** 10.1186/s13018-017-0566-3

**Published:** 2017-04-21

**Authors:** Dong Ren, Yueju Liu, Xianchao Zhang, Zhaohui Song, Jian Lu, Pengcheng Wang

**Affiliations:** 1 Third Hospital of Hebei Medical University, Shijiazhuang, 050051 China; 2 Hebei Provincial Key Laboratory of Orthopaedic Biomechanics, Shijiazhuang, 050051 Hebei China; 3 Department of Orthopedic Center, Third Hospital of Hebei Medical University, 139 Zi Qiang Road, Shijiazhuang, 050051 Hebei China

**Keywords:** Biomechanics, Finite element, Knee joint, Medial collateral ligament, Model

## Abstract

**Background:**

A three-dimensional finite element model (FEM) of the knee joint was established to analyze the biomechanical functions of the superficial and deep medial collateral ligaments (MCLs) of knee joints and to investigate the treatment of the knee medial collateral ligament injury.

**Methods:**

The right knee joint of a healthy male volunteer was subjected to CT and MRI scans in the extended position. The scanned data were imported into MIMICS, Geomagic, and ANSYS software to establish a three-dimensional FEM of the human knee joint. The anterior-posterior translation, valgus-varus rotation, and internal-external rotation of knee joints were simulated to observe tibial displacement or valgus angle. In addition, the magnitude and distribution of valgus stress in the superficial and deep layers of the intact MCL as well as the superficial, deep, and overall deficiencies of the MCL were investigated.

**Results:**

In the extended position, the superficial medial collateral ligament (SMCL) would withstand maximum stresses of 48.63, 16.08, 17.23, and 16.08 MPa in resisting the valgus of knee joints, tibial forward displacement, internal rotation, and external rotation, respectively. Meanwhile, the maximum stress tolerated by the SMCL in various ranges of motion mainly focused on the femoral end point, which was located at the anterior and posterior parts of the femur in resisting valgus motion and external rotation, respectively. However, the deep medial collateral ligament could tolerate only minimum stress, which was mainly focused at the femoral start and end points.

**Conclusions:**

This model can effectively analyze the biomechanical functions of the superficial and deep layers of the MCLs of knee joints. The results show that the knee MCL II° injury is the indication of surgical repair.

## Background

The medial collateral ligament (MCL) plays an important role in limiting and maintaining the movement of the knee joint and protecting its stability [1]. There is a high incidence of injury to the knee MCL in sports activities such as ice hockey, skiing, and soccer [2], accounting for approximately 40% of all severe knee joint injuries, 50% of which involve partial fracture while 30% involve complete fracture and injury of the knee MCL [3]. These injuries may ultimately lead to medial laxity and instability of the knee joints, as well as secondary long-term complications. Most surgeons [4] advocate conservative treatment for the knee MCL I° injury and surgical repair for the knee MCL III° injury, respectively. However, the option to deal with MCL II° injury is controversial. This study is to evaluate the function in detail within MCL maintaining the stability of the knee joint and expects to provide evidence on how to treat the knee MCL II° injury.

## Methods

### General information

A healthy male volunteer (age, 27 years; height, 177 cm; weight, 75 kg) without any right knee deformity, history of trauma, or clinically positive signs was selected for the study. He consented to participate in this test by signing an informed consent.

### Acquisition of CT and MR imaging data

The right knee joint of the volunteer was subjected to continuous spiral CT in a relaxation and extended position, from 95 mm above the upper margin of the patella to 110 mm below the knee joint line, i.e., from the middle lower segment of the femur to the middle upper segment of the tibiofibula. The scan parameters were as follows: layer thickness of 0.7 mm, matrix size of 512 × 512, and pixel size of 0.705 mm; in total, 369 Digital Imaging and Communications in Medicine (DICOM)-format images were acquired.

MR imaging was performed for the same right knee joint in the same position, from 50 mm above the upper margin of the patella to 70 mm below the knee joint line, in which the axial T1W1 sequence was selected. The scan parameters were as follows: TR of 1900 ms, TE of 2.58 ms, layer thickness of 1 mm, matrix size of 256 × 256, and pixel size of 0.859 mm; a total of 176 DICOM-format images were obtained.

### Establishment of bone tissue model of knee joints based on CT images

The obtained CT data were imported into an interactive medical image control system, Materialise Interactive Medical Image Control System (MIMICS) 14.0 (Materialise, Leuven, Belgium). A three-dimensional model of the original bone tissue of the knee joint was obtained using the threshold segmentation and three-dimensional model calculation and was imported into automatic reverse engineering software, Geomagic Studio 12.0 (Geomagic, USA), for optimization, so as to obtain a finer bone tissue model. The model was again imported into MIMICS 14.0 software, which was initially meshed in the 3-matic module, and the 4-node tetrahedral element was transformed into a 10-node tetrahedral element.

### Establishment of ligament and meniscus models based on MR images

The method was basically the same as mentioned above, except for the following aspects: (1) Due to the unclear boundary between the soft tissues in the MIMICS 14.0 workspace, individual planes of the meniscus and ligaments were required to be split manually, followed by calculation to obtain the original meniscus and ligament models of the knee joints. (2) In some MCLs, differentiating the superficial and deep layers was difficult; they required to be separated using the trimmer, stretching, Boolean subtraction, and other functions in Geomagic Studio 12.0 according to their length, width [5], thickness ratio, and differences in their other normal anatomic structures, by obtaining their fine models. (3) Before ligament and meniscus models were initially meshed in the 3-matic module of MIMICS 14.0 software, they were subjected to Boolean subtraction calculation in Geomagic Studio 12.0 software to obtain the three-dimensional models.

### Finite element partition and analysis

#### Model assembly

**Fig. 1:**
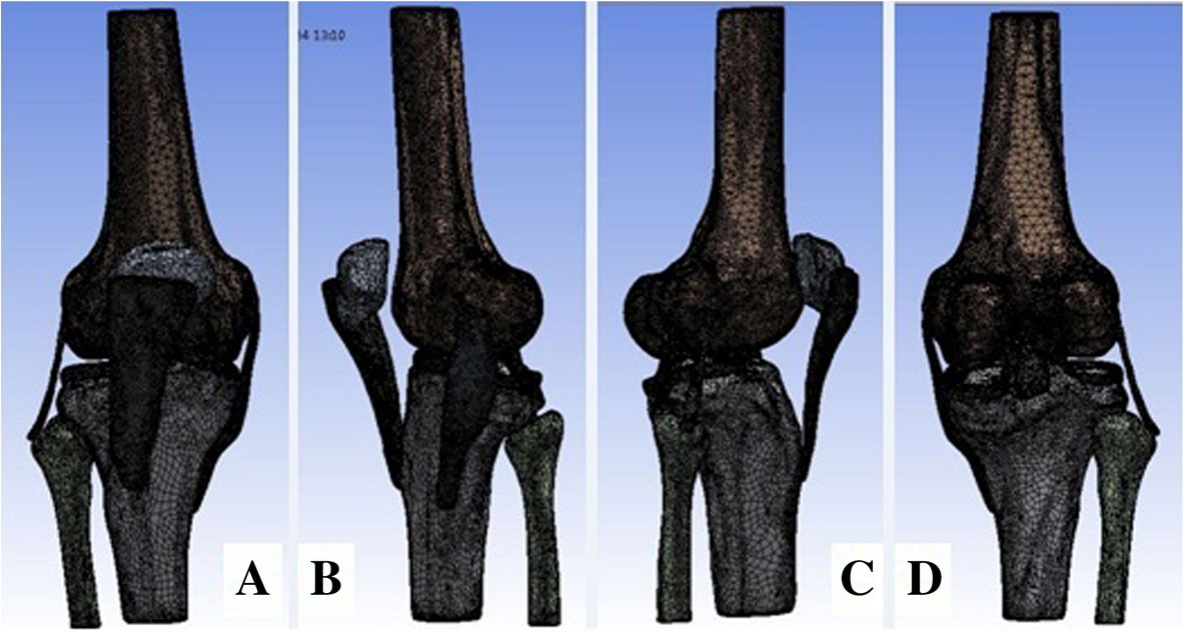
Mesh generation in ANSYS Workbench. **a** Anterior view. **b** Medial view. **c** Lateral view. **d** Posterior view

Bone tissues, ligaments, and meniscus models were saved in cdb format and imported into the workbench of ANSYS 13.0 software (ANSYS, USA). The models were then assembled, and material properties were applied as per the properties reported in the literature [6, 7] (Table 1). Contact of the starting and ending points of each ligament with the bones, and that of the superficial and deep layers of the medial collateral ligament with the meniscus were defined as bonded contact, while contacts at other sites were defined as “no separation contacts.” The models were remeshed using an interactive mesh of pentahedral and hexahedral elements, and a total of 877,070 nodes and 354,003 elements were obtained, as shown in Fig. 1.

**Table 1:** Material parameters of the normal finite element model

Structure	*E* (MPa)	*V*
Femur	3883.4	0.3
Tibia	4184.6	0.3
Fibula	–	0.3
Patella	–	0.3
Menisci	59	0.3
ACL	1.046	0.4
PCL	1.035	0.4
SMCL	1.063	0.4
DMCL	1.063	0.4
LCL	1.063	0.4
PL	1.035	0.4

*ACL* anterior cruciate ligament, *DMCL* deep medial collateral ligament, *E* Young’s modulus, *LCL* lateral collateral ligament, *PCL* posterior cruciate ligament, *PL* patellar ligament, *SMCL* superficial medial collateral ligament, *V* Poisson’s ratio

#### Loads and boundaries

The upper femur was fixed in 6 degrees of freedom (DOF), and 134-N forward force, 134-N backward force, 10-N m valgus torque, and 10-N m external rotation torque and internal rotation torque were applied to the femur.

#### Calculation and post processing

**Fig. 2:**
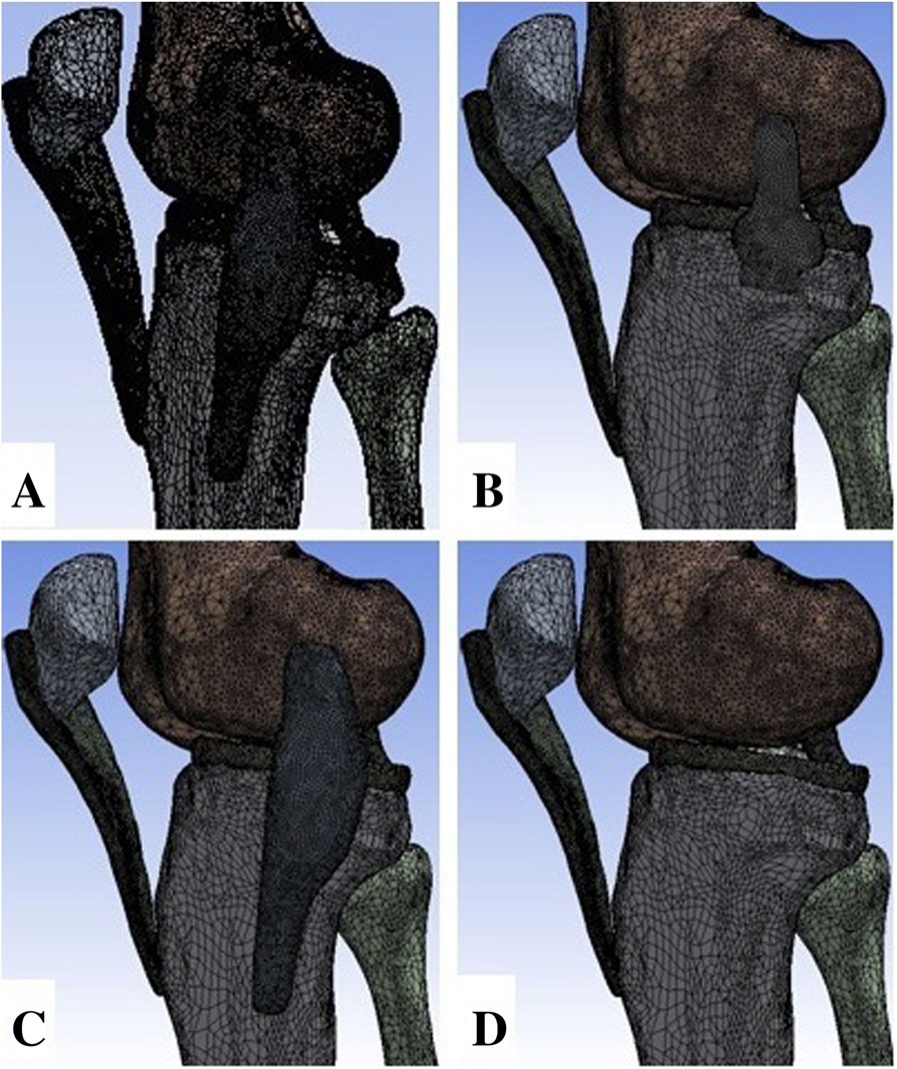
Four cases of MCL deficiency. **a** Case 1: intact MCL. **b** Case 2: SMCL deficiency. **c** Case 3: DMCL deficiency. **d** Case 4: overall deficiencies of the MCL

The tibial displacement or valgus angle as well as the stress magnitude and distribution in the superficial and deep layers of medial collateral ligaments under conditions of intact MCL (case 1) as well as superficial MCL (SMCL) deficiency (case 2), deep MCL (DMCL) deficiency (case 3), and overall deficiencies of the MCL (case 4) is described in Fig. 2.

#### Model validation

The tibial anterior translation was observed to be 4.89 mm when constraining the 6 DOF in the upper femur and applying a forward force of 134 N to the tibia in an extension position of the knee joint; the translation was reported to be 4.6–5.0 mm using the same load in previous studies [8]. Thus, our results were consistent with the previously reported results on FEM studies, suggesting the effectiveness of our model.

## Results

**Fig. 3:**
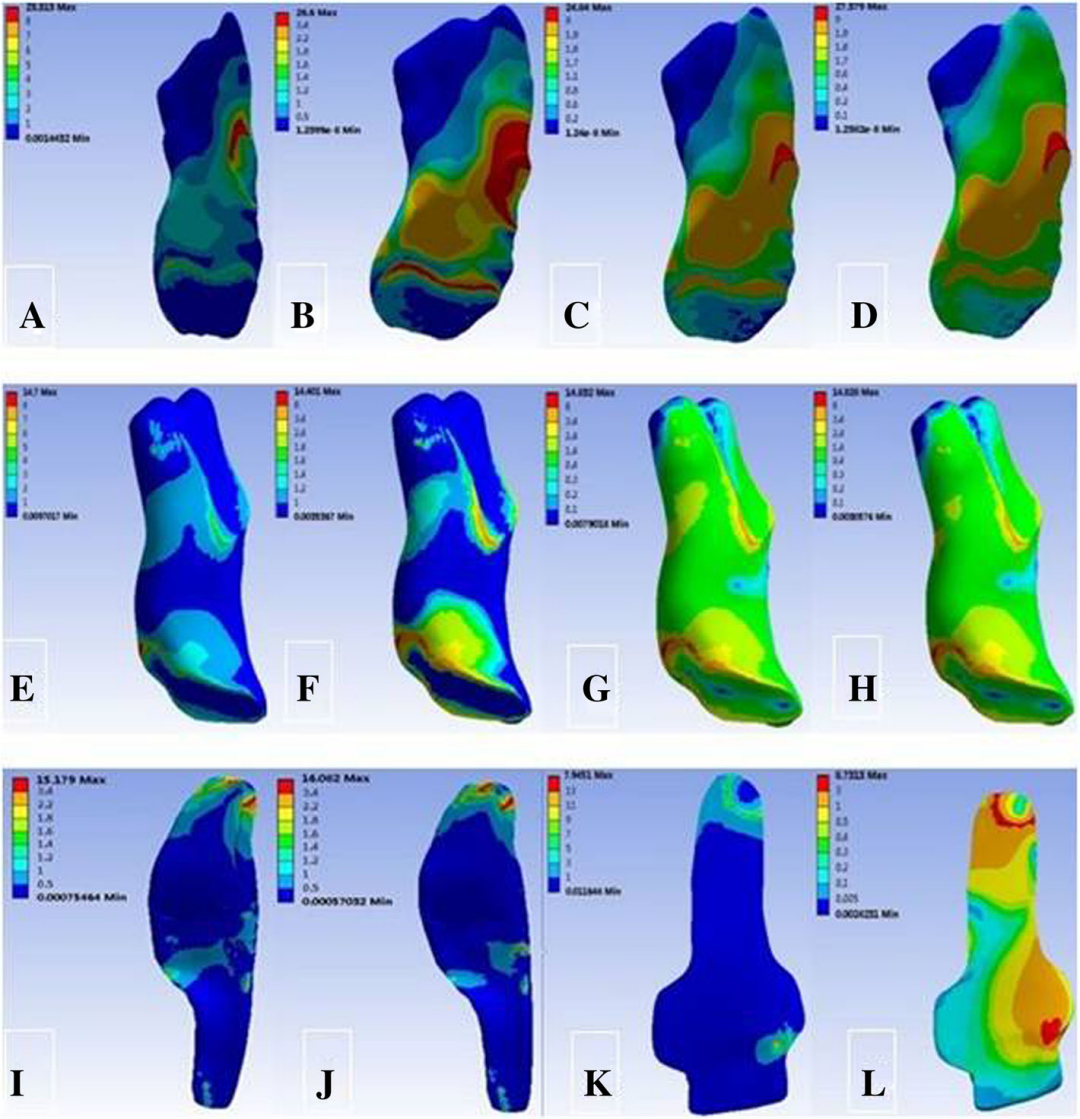
von Mises stress distribution of the ACL, PCL, SMCL, and DMCL under a force of 134 N in anterior translation. **a** ACL in case 1. **b** ACL in case 2. **c** ACL in case 3. **d** ACL in case 4. **e** PCL in case 1. **f** PCL in case 2. **g** PCL in case 3. **h** PCL in case 4. **i** SMCL in case 1. **j** SMCL in case 3. **k** DMCL in case 1. **l** DMCL in case 2

Under the load of the 134-N forward force, the tibial displacement changed from 4.89 mm at intact MCL to 5.17, 5.04, and 5.17 mm at SMCL deficiency, DMCL deficiency, and overall MCL deficiency, respectively. The peak stress was maximum at the anterior cruciate ligament (ACL), lower at SMCL, and minimum at DMCL (Table 2) and was mainly located at the femoral end point at both ACL and SMCL and at the start and end points at DMCL (Fig. 3).

**Table 2:** Response parameters of the knee joint under a force of 134 N in anterior translation

	Tibial displacement (mm)	Peak stress (MPa)			
		ACL	PCL	SMCL	DMCL
Case 1	4.89	23.31	14.70	15.18	7.95
Case 2	5.17	26.60	14.40	–	8.73
Case 3	5.04	24.84	14.83	16.08	–
Case 4	5.27	27.38	14.83	–	–

*ACL* anterior cruciate ligament, *DMCL* deep medial collateral ligament, *PCL* posterior cruciate ligament, *SMCL* superficial medial collateral ligament

**Fig. 4:**
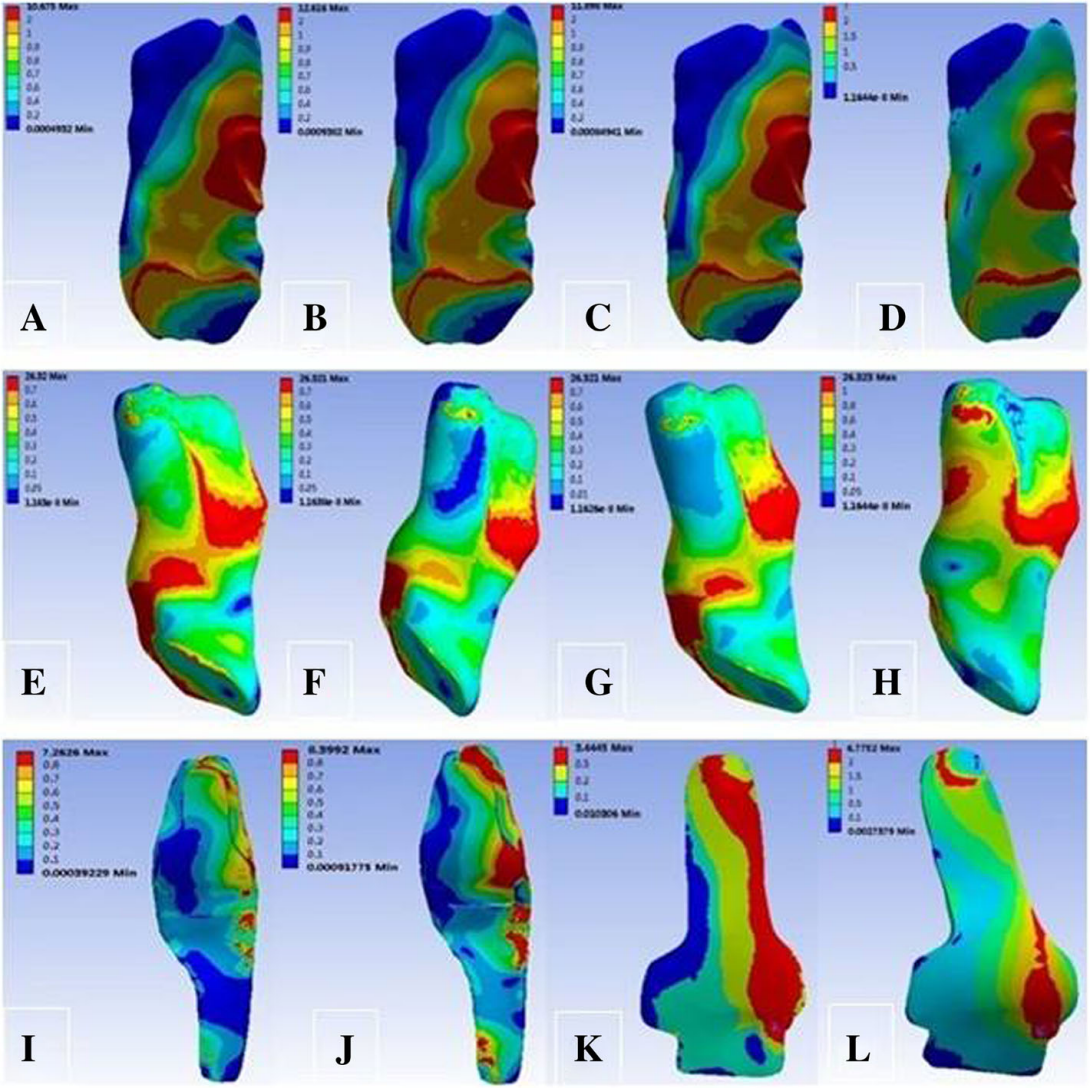
von Mises stress distribution of ACL, PCL, SMCL, and DMCL under a force of 134 N in posterior translation. **a** ACL in case 1. **b** ACL in case 2. **c** ACL in case 3. **d** ACL in case 4. **e** PCL in case 1. **f** PCL in case 2. **g** PCL in case 3. **h** PCL in case 4. **i** SMCL in case 1. **j** SMCL in case 3. **k** DMCL in case 1. **l** DMCL in case 2

Under the load of the 134-N backward force, the tibial displacement changed from 4.98 mm at intact MCL to 4.99, 4.92, and 5.02 mm at SMCL deficiency, DMCL deficiency, and overall MCL deficiency, respectively. The peak stress was maximum at the posterior cruciate ligament (PCL), lower at SMCL, and very low at DMCL (Table 3) and was mainly located at the femoral start and end points at PCL and DMCL and only at the femoral end point at SMCL (Fig. 4).

**Table 3:** Response parameters of the knee joint under a force of 134 N in posterior translation

	Tibial displacement (mm)	Peak stress (MPa)			
		ACL	PCL	SMCL	DMCL
Case 1	4.98	10.68	26.32	7.26	3.44
Case 2	4.99	12.62	26.32	–	6.78
Case 3	4.92	11.90	26.32	8.40	–
Case 4	5.02	12.72	26.32	–	–

*ACL* anterior cruciate ligament, *DMCL* deep medial collateral ligament, *PCL* posterior cruciate ligament, *SMCL* superficial medial collateral ligament

**Fig. 5:**
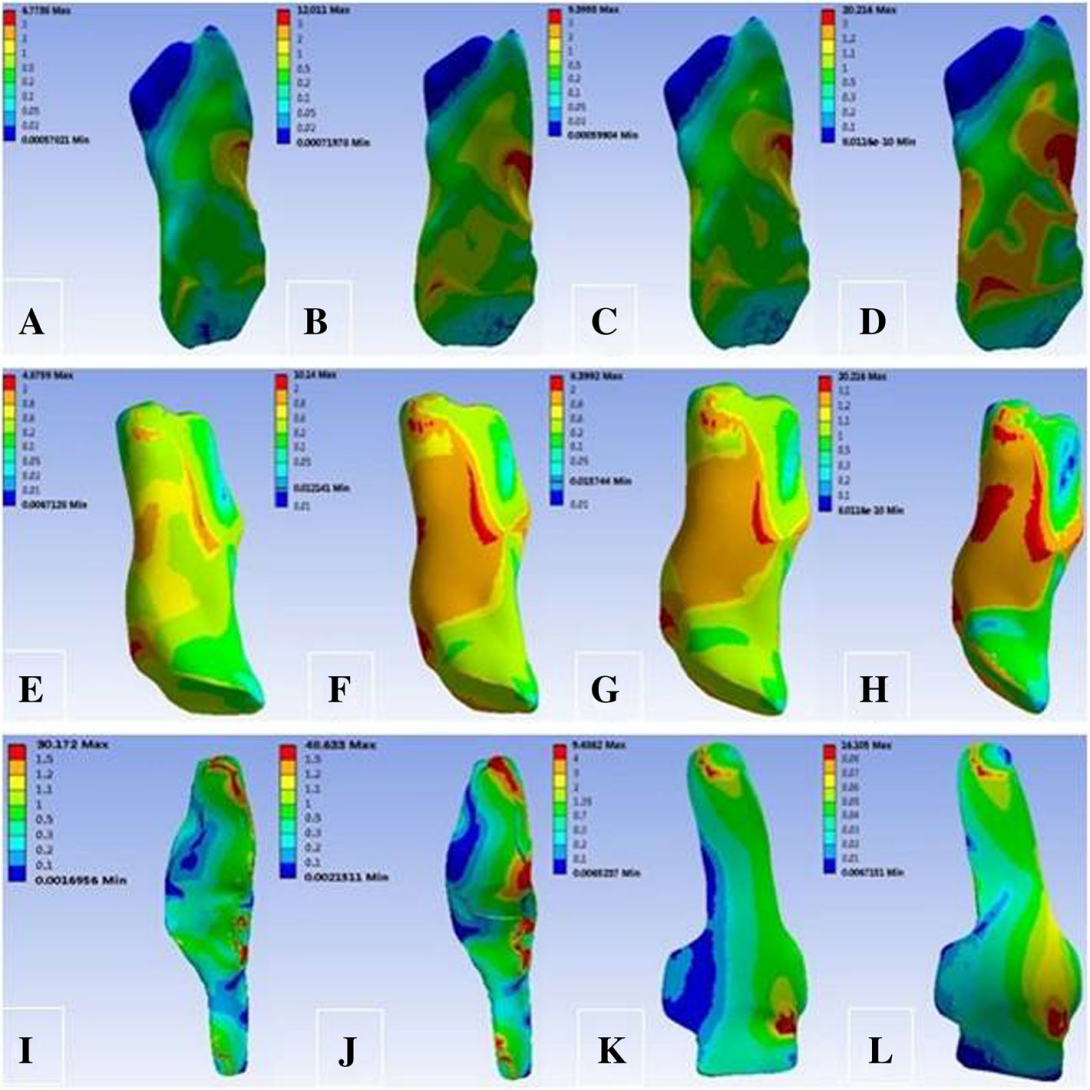
von Mises stress distribution of ACL, PCL, SMCL, and DMCL under 10 N m of valgus moment. **a** ACL in case 1. **b** ACL in case 2. **c** ACL in case 3. **d** ACL in case 4. **e** PCL in case 1. **f** PCL in case 2. **g** PCL in case 3. **h** PCL in case 4. **i** SMCL in case 1. **j** SMCL in case 3. **k** DMCL in case 1. **l** DMCL in case 2

Under the load of the 10-N m valgus torque, the tibial valgus angle changed from 4.06° at intact MCL to 6.08°, 4.86°, and 6.22° at SMCL deficiency, MCL deficiency and overall MCL deficiency, respectively. The peak stress was maximum at SMCL and gradually decreased at DMCL, ACL, and PCL (Table 4); it was mainly located at the femoral end point and anterior part at SMCL and at the femoral start and end points at DMCL (Fig. 5).

**Table 4:** Response parameters of the knee joint under 10 N m of valgus moment

	Tibial valgus angle (°)	Peak stress (MPa)			
		ACL	PCL	SMCL	DMCL
Case 1	4.06	6.77	4.88	30.17	9.49
Case 2	6.08	12.01	10.14	–	16.11
Case 3	4.86	9.40	8.40	48.63	–
Case 4	6.22	20.22	20.22	–	–

*ACL* anterior cruciate ligament, *DMCL* deep medial collateral ligament, *PCL* posterior cruciate ligament, *SMCL* superficial medial collateral ligament

**Fig. 6:**
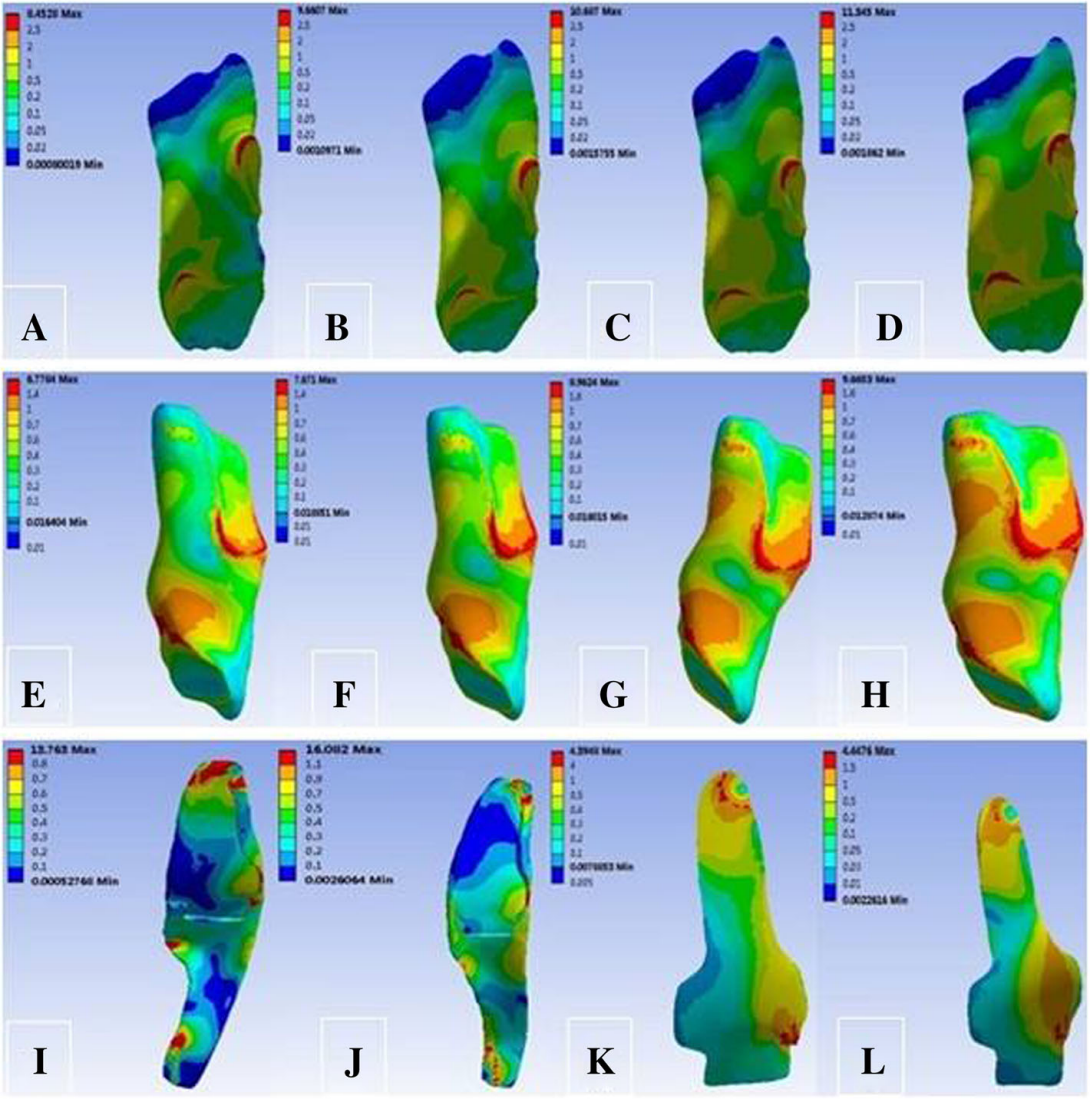
von Mises stress distribution of the ACL, PCL, SMCL, and DMCL under 10 N m of external rotation moment. **a** ACL in case 1. **b** ACL in case 2. **c** ACL in case 3. **d** ACL in case 4. **e** PCL in case 1. **f** PCL in case 2. **g** PCL in case 3. **h** PCL in case 4. **i** SMCL in case 1. **j** SMCL in case 3. **k** DMCL in case 1. **l** DMCL in case 2

Under the load of the 10-N m external rotation torque, the tibial external rotation angle changed from 5.92° at intact MCL to 5.95°, 5.94°, and 6.10° at SMCL deficiency, DMCL deficiency, and overall MCL deficiency, respectively. The peak stress was maximum at SMCL, relatively lower at ACL and PCL, and the lowest at DMCL (Table 5). The peak stress at SMCL was mainly located at the end point and posterior part of the femur, and that at DMCL was located at the start and end points (Fig. 6).

**Table 5:** Response parameters of the knee joint under 10 N m of external rotation moment

	Tibial external rotation angle (°)	Peak stress (MPa)			
		ACL	PCL	SMCL	DMCL
Case 1	5.92	8.45	6.79	13.76	4.39
Case 2	5.95	9.66	7.67	–	4.45
Case 3	5.94	10.69	8.96	16.08	–
Case 4	6.10	11.35	9.67	–	–

*ACL* anterior cruciate ligament, *DMCL* deep medial collateral ligament, *PCL* posterior cruciate ligament, *SMCL* superficial medial collateral ligament

**Fig. 7:**
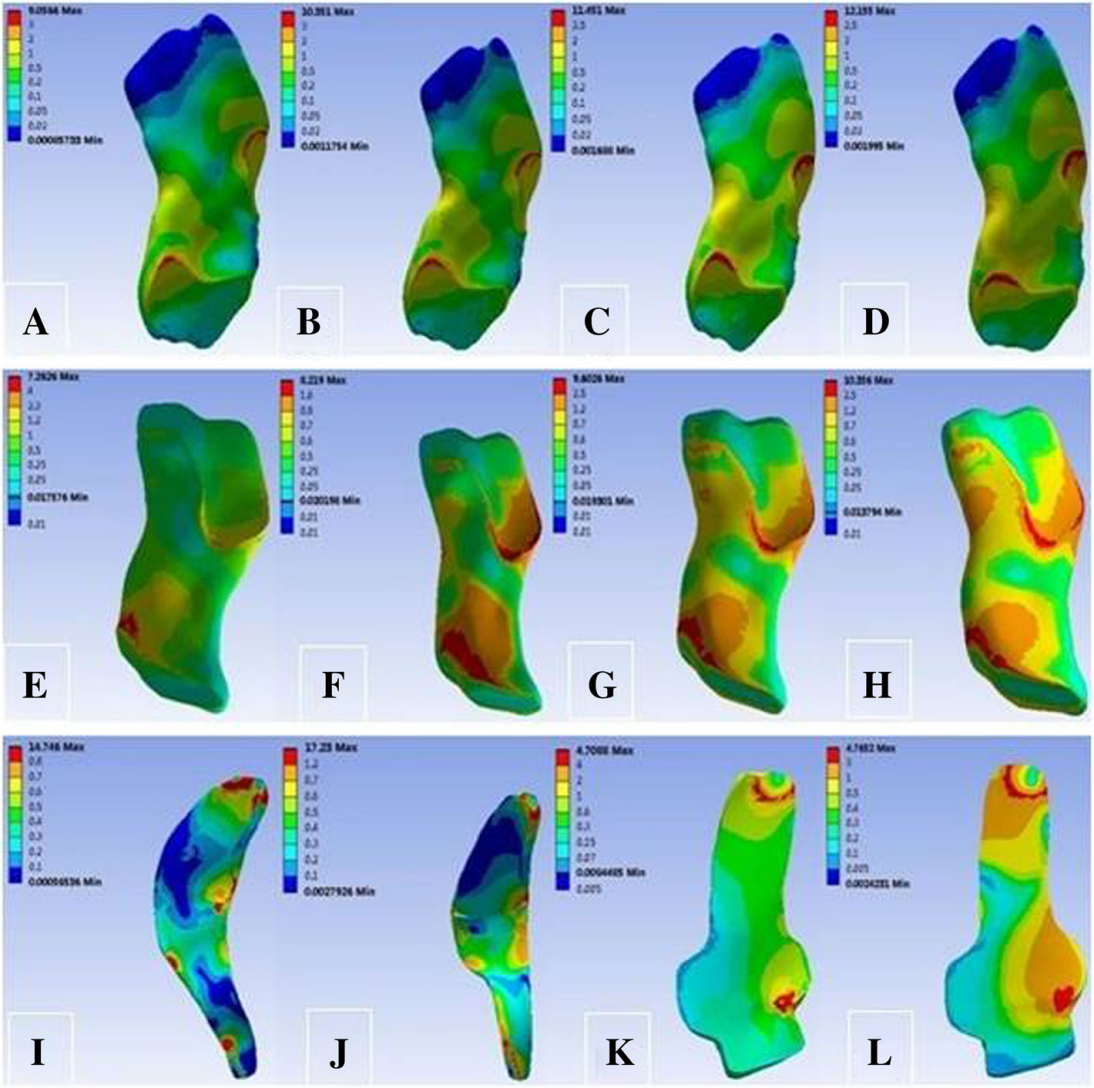
von Mises stress distribution of the ACL, PCL, SMCL, and DMCL under 10 N m of internal rotation moment. **a** ACL in case 1. **b** ACL in case 2. **c** ACL in case 3. **d** ACL in case 4. **e** PCL in case 1. **f** PCL in case 2. **g** PCL in case 3. **h** PCL in case 4. **i** SMCL in case 1. **j** SMCL in case 3. **k** DMCL in case 1. **l** DMCL in case 2

Under the load of the 10-N m internal rotation torque, the tibial internal rotation angle changed from 6.64° at intact MCL to 7.48°, 6.72°, and 7.57° at SMCL deficiency, DMCL deficiency, and overall MCL deficiency, respectively. Meanwhile, the peak stress was maximum at SMCL, relatively lower at ACL and PCL, and minimum at DMCL (Table 6) and was mainly located at the femoral end point at SMCL and at the femoral start and end points at DMCL (Fig. 7).

**Table 6:** Response parameters of the knee joint under 10 N m of internal rotation moment

	Tibial internal rotation angle (°)	Peak stress (MPa)			
		ACL	PCL	SMCL	DMCL
Case 1	6.64	9.06	7.26	14.75	4.71
Case 2	7.48	10.35	8.22	–	4.77
Case 3	6.72	11.45	9.60	17.23	–
Case 4	7.57	12.16	10.36	–	–

*ACL* anterior cruciate ligament, *DMCL* deep medial collateral ligament, *PCL* posterior cruciate ligament, *SMCL* superficial medial collateral ligament

## Discussion

### Establishment of the knee joint model

The knee joint is one of the most complex joints in the human body, with a complex anatomic structure and biomechanical properties. The traditional mechanical method to study its biomechanical functions usually involves the application of extra-articular loads and use of mechanical measuring instruments [9], and it is difficult to investigate the stress distribution within the joints and other issues using this method. Therefore, establishing knee joint models such as the crossed four-link physical model and the two-dimensional mathematical model of the sagittal knee joint as well as the three-dimensional model of dynamic response of the knee joint has become an important measure for further studying the biomechanical characteristics of knee joints [10]. Since its first application in orthopedic biomechanics by Brekelmans et al. [11] in 1972, the FEM has been widely used in modeling teeth, artificial limbs, spine, etc. [12] and has been gradually applied to the biomechanics of ankles, knees, wrists, and other joints [13, 14].

Single-mode CT or MR images are typically unable to provide a clear contrast for intact knee joints, leading to difficulty in accurately constructing an FEM of knee joints containing multiple anatomic structures. Studies have found that although CT image data alone can be used to accurately construct bone structure models, it cannot be used to accurately simulate the cartilage, ligament, meniscus, and other soft tissues [15]. In contrast, MR imaging data alone can be used to accurately construct the anatomic structure models of various soft tissues including knee joints, while it cannot be used to accurately simulate bone structures [16]. Thus, using CT or MR imaging alone will significantly decrease the accuracy of these models, leading to inaccurate mechanical analysis of the knee joints.

Yao et al. [17] accurately constructed FEMs of the femoral cartilage, tibial cartilage, and medial meniscus using MATLAB and Hypermesh software, but not of other structures of knee joints. Therefore, a single software often has some limitations, and the constructed FEM fails to truly represent the anatomic characteristics of knee joints; thus, the FEMs of knee joints can be accurately constructed only by collaborative application of a variety of modeling software.

In this study, a variety of modes of CT and MR imaging as well as MIMICS 14.0, Geomagic Studio 12.0, and ANSYS modeling software were applied to construct a three-dimensional FEM of knee joints using the reverse engineering (RE) principle. In the MIMICS software, the structures in CT and MR images were assembled according to the human anatomy. However, this assembled model was very coarse due to the presence of interference surfaces. This issue could be addressed using Geomagic Studio 12.0, in which the interference surfaces in the model were removed. However, this software was not able to generate ANSYS pre-processing files; therefore, the repaired models were imported again into the 3-matic module of MIMICS software for initial meshing before being imported into ANSYS for finite element analysis (FEA). In addition, since this study focused on the mechanical analyses of the ligaments, their mesh size was refined at 1 mm, and the bone structures were set as a solid body with a mesh size of 4 mm, reflecting the different focuses of subjects in FEA. Because the meshing quality determined the accuracy of the FEA results, an even finer mesh would be required to analyze non-linear contacts. Local meshes with poor quality were optimized, and interactive meshes with satisfactory 6-node pentahedrons and 8-node hexahedrons were obtained based on the high-quality area meshes, in order to achieve more accurate results compared to those obtained using meshes with 10-node tetrahedron elements. Ultimately, a three-dimensional model of the human right knee joint containing a variety of anatomic structures, such as the middle and upper segments of the femur, middle and upper segments of the tibia, fibula, patella, meniscus, ACL, PCL, MCL, lateral collateral ligament, and patellar ligament, was constructed. Meanwhile, high-quality volume meshes were developed, satisfying the requirements for FEA of biomechanics of knee joints. This FEM can be used to analyze the stress distribution of ligaments, contacts of tibiofemoral joints, stress distribution on articular surface, changes of stress distribution under different ligament deficiencies, and other biomechanical studies, as well as to simulate the effects of surgical results on the biomechanics of knee joints under different surgical conditions, and conduct biomechanical analyses of surgical fixations.

### Biomechanical analyses of medial collateral ligament of the knee joint

The anatomy of MCL has been extensively studied [5]. In this experiment, based on the human anatomy, an FEM of the knee joint was established to simulate the anterior-posterior translation, valgus-varus rotation, and internal-external rotation of the knee joint, so as to study the biomechanical functions of its superficial and deep MCLs, in which the knee joint varus was excluded because the knee MCL is completely relaxed in this condition. In the experiment, a gradually increasing color grading from blue to red color indicated gradually increasing von Mises stress, which represented a greater load on the ligament and a greater role of the site and likelihood of damage.

Under the load of the 134-N forward force, the tibial displacement changed from 4.89 mm at intact MCL to 5.17, 5.04, and 5.17 mm at SMCL deficiency, DMCL deficiency, and overall MCL deficiency, respectively. A greater variation of tibial displacement at overall MCL deficiency indicated that MCL plays a role in limiting the forward translation of the tibia. Meanwhile, the tibial displacement showed a greater variation at SMCL deficiency compared with that at DMCL deficiency, suggesting that SMCL has a greater effect than the DMCL. During this process, the stress at ACL maintained a maximum value, suggesting that ACL plays the most important role in limiting the tibial anterior translation. Moreover, a greater stress at SMCL than that at DMCL indicated that the SMCL has a greater effect. The stress nephogram showed that the peak stresses at ACL and SMCL were mainly located at the femoral end point, indicating that during tibial anterior translation, injury to the femoral end point is most likely to occur at ACL and SMCL, and less likely to occur at DMCL.

Under the load of the 134-N backward force, the tibial translation showed a very small variation with MCL deficiency, during which the stress at PCL maintained a maximum value, while the stresses at SMCL and DMCL were relatively small, suggesting that PCL plays the most important role in constraining the tibial posterior translation, while the effects of SMCL and DMCL are very small. Meanwhile, the peak stress at PCL occurred at the tibial start and end points, suggesting that in tibial posterior translation, injury is most likely to occur at the femoral start and end points at PCL, while the risk of injury at SMCL and DMCL is small.

Under the load of the 10-N m valgus torque, the tibial valgus angle showed a large variation with MCL deficiency, which changed from 4.06° at intact MCL to 6.08°, 4.86°, and 6.22° at SMCL deficiency, MCL deficiency, and overall MCL deficiency, respectively, suggesting that MCL tends to resist the valgus motion of knee joints. Meanwhile, the stress was the largest at SMCL followed by that at DMCL, indicating that SMCL plays the most important role in limiting the valgus motion and the effect of DMCL is relatively smaller. As evident in the stress nephogram, the peak stress at SMCL occurred at the end point and anterior part of the femur, indicating that injury is most likely to occur at the end point and anterior part of the femur in valgus motion of knee joints at SMCL. In contrast, the peak stress at DMCL occurred at the femoral start and end points, suggesting that they are prone to injury at DMCL.

Under the load of the 10-N m external rotation torque, the tibial external rotation angle changed from 5.92° at intact MCL to 5.95°, 5.94°, and 6.10° at SMCL deficiency, DMCL deficiency, and overall MCL deficiency, respectively. The tibial external rotation angle showed a large variation at overall MCL deficiency, suggesting that MCL tends to resist the external rotation of the knee joints. Although the tibial external rotation angle did not show significant difference between SMCL and DMCL deficiencies, the stress at SMCL was larger than that at DMCL, indicating that SMCL plays a more significant role in limiting the external rotation of the knee joint than the DMCL. As observed in the stress nephogram, the peak stress at SMCL was mainly located at the femoral end point and posterior part, indicating that they are prone to injury at SMCL during external rotation of knee joints, while the injury at DMCL was smaller.

Under the load of the 10-N m internal rotation torque, the tibial internal rotation angle changed from 6.64° at intact MCL to 7.48°, 6.72°, and 7.57° at SMCL deficiency, DMCL deficiency, and overall MCL deficiency, respectively. The tibial internal rotation angle showed a larger variation than the tibial external rotation angle with MCL deficiency, suggesting that knee joint MCL has a greater effect on limiting the internal rotation than the external rotation. Similarly, greater stress at SMCL than that at DMCL indicated that the SMCL has a greater effect on limiting the internal rotation of knee joints than the DMCL. As observed in the stress nephogram, the peak stress at SMCL occurred at the femoral end point, indicating that the femoral end point was prone to injury at SMCL during internal rotation of knee joints, while the injury at DMCL was smaller.

The above analyses show that in the extended position of knee joints, the main effect of MCL is to resist the valgus motion of knee joints, along with limiting the tibial forward displacement as well as the internal and external rotations of knee joints. The SMCL plays the most important role in the structure of the MCL of knee joints, while the effects of DMCL are relatively lesser. In various motions of knee joints, the femoral end point at SMCL is the most prone to injury. The anterior part of the femur is more prone to injury in resisting valgus motion, and the posterior part in resisting external rotation at SMCL. However, injury is less likely to occur at DMCL, and when it does occur, it occurs at the femoral start and end points.

## Conclusions

In summary, this model to evaluate the function of the MCL by FEA is reliable [18–20]. The results indicate that the knee MCL II° injury should be repaired by surgery. However, the shortcoming of this study is the lack of clinical evidence. We hope to achieve a further investigation in the clinic.

## Abbreviations

ACL: Anterior cruciate ligament
CT: Computed tomography
DICOM: Digital Imaging and Communications in Medicine
DMCL: Deep medial collateral ligament
*E*: Young’s modulus
FEA: Finite element analysis
FEM: Finite element model
LCL: Lateral collateral ligament
MCL: Medial collateral ligament
MIMICS: Materialise Interactive Medical Image Control System
MRI: Magnetic resonance imaging
PCL: Posterior cruciate ligament
PL: Patellar ligament
RE: Reverse engineering
SMCL: Superficial medial collateral ligament
*V*: Poisson’s ratio
